# Engineering S-scheme Ag_2_CO_3_/g-c_3_N_4_ heterojunctions sonochemically to eradicate Rhodamine B dye under solar irradiation

**DOI:** 10.1039/d3ra00173c

**Published:** 2023-04-19

**Authors:** Ali Alsulmi, Mohamed H. Shaker, Abanoob M. Basely, M. F. Abdel-Messih, Ayman Sultan, M. A. Ahmed

**Affiliations:** a Department of Chemistry, College of Science, King Saud University P.O.2455 Riyadh 11451 Saudi Arabia; b Chemistry Department, Faculty of Science, Ain Shams University Egypt abdelhay71@hotmail.com; c Department of Chemistry, University of York York YO10 5DD UK

## Abstract

The use of natural solar radiation is a low-cost significant technology for water pollution remediation and production of clean energy. In this work, S-scheme Ag_2_CO_3_/g-C_3_N_4_ heterojunctions were engineered for carefully eradicating Rhodamine B dye under natural sunlight irradiation. Solid thermal decomposition reactions generate g-C_3_N_4_ sheets by annealing urea at 520 °C. Ag_2_CO_3_ nanoparticles are directed and localized sonochemically to the active centers of g-C_3_N_4_ sheets. The physicochemical properties of the solid specimen were determined by PL, DRS, XRD, HRTEM, mapping, EDX, N_2_-adsorption–desorption isotherm and XPS analyses. As elucidated by HRTEM, PL and DRS analyses, 5 wt% of spherical Ag_2_CO_3_ nanoparticles deposited on the g-C_3_N_4_ sheet surface and nearly equidistant from each other elevate the electron–hole separation efficiency and broaden the absorption capacity of photocatalysts. Rhodamine B dye was degraded at a rate of 0.0141 min^−1^ by heterojunctions containing 5 wt% Ag_2_CO_3_ and 95 wt% g-C_3_N_4_, which is three-fold higher than that on pristine g-C_3_N_4_ nanosheets. Free radical scrubber experiments revealed the contribution of charge carriers and reactive oxygen species to the decomposition of RhB dye with a preferential role of positive holes and superoxide species. PL measurements of terephthalic acid and scrubber trapping experiments provide confirmatory evidence for charge diffusion *via* the S-scheme mechanism that accounts for the production of electron–hole pairs with strong redox power. This novel research work is contributory to manipulate the S-scheme heterojunction for efficient and low-cost wastewater treatment under natural solar irradiation.

## Abstract 2

The use of natural solar radiation is a low-cost significant technology for water pollution remediation and production of clean energy.

## Graphical abstract

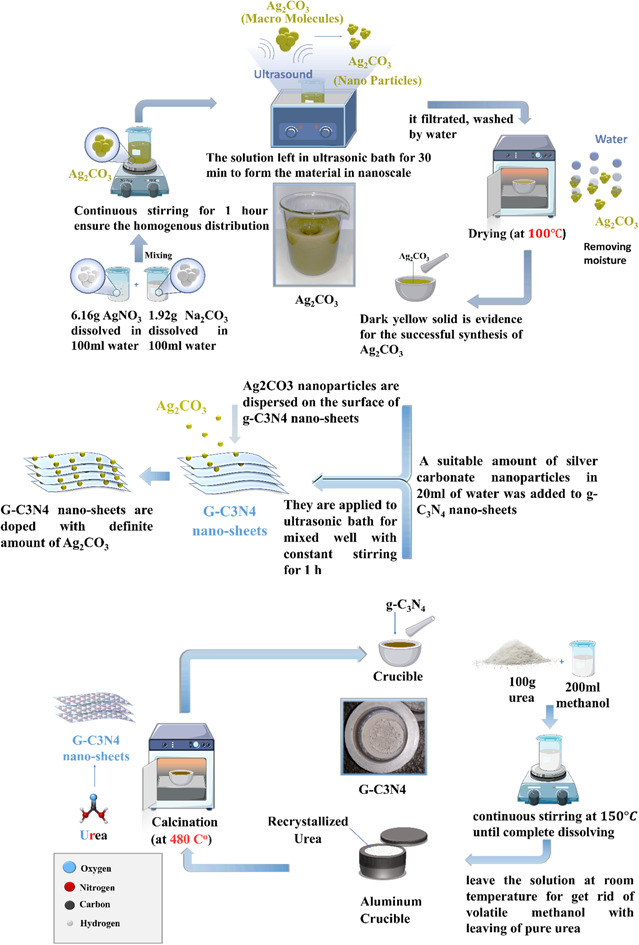

## 1. Introduction

Water pollution is an environmental crisis that urgently needs to be addressed to meet the increasing demand for fresh water. Organic dyes are the major toxic pollutants discharged from different industries causing serious problems to human health and aquatic life.^1–4^ Adsorption, reverse osmosis, electrochemical process, coagulation, enzymatic treatment and ion-exchange processes are very expensive, complicated techniques that produce a second generation of pollutant materials.^5–10^ Photocatalysis on the surface of semiconductors is a green technology for the destruction of organic wastes into eco-friendly species. The photocatalytic reaction requires two factors to achieve optimization conditions. The first one is a low-cost and broadband solar radiation source that is sufficient to decompose millions of molecules of pollutants. The second requirement is an appropriate semiconductor with tunable band gap energy and extremely high surface area that generates huge amounts of reactive oxygen species, which can destruct toxic pollutants in a short time under light irradiation. Natural sunlight in our country is very abundant in all seasons, particularly in summer days and acts as a low-cost radiation source. The natural sunlight contains a minor amount of UV radiation (5%), visible radiation (43%) and near infra-red (NIR) radiation (50%). Most of the previous research studies have focused on investigating the photocatalytic process under UV and visible light irradiation. In recent years, NIR is very effective in decomposing organic pollutants *via* photocatalytic and thermal routes. An appropriate semiconductor from the perspective of thermodynamics and kinetics must have the advantages of low cost, environmentally benign nature, high separation rate and fast transfer of charge carriers. Efficient semiconductors with wide bandgap structures as TiO_2_, SnO_2_, ZnO and CeO_2_ show high rate of organic pollutant decomposition^11–16^ under UV irradiation that constitutes only 5% of the natural solar radiation. Narrow band gap energy semiconductors absorb visible light radiation; however, the ultra-fast electron–hole pair coulombic attraction decreased the life time required to generate reactive oxygen species.^17–20^ In conclusion, a single semiconductor cannot achieve the optimization properties for decomposing the organic pollutants. To overcome the limitations of single photocatalysts, heterojunctions composed of wide band gap energy with high efficiency for charge carrier separation and narrow band gap energy that harvests solar radiation are more favored. Recently, graphitic carbon nitride (g-C_3_N_4_), has gained great interest in photocatalytic reactions due to its good stability in basic and acidic media, low synthesis costs, non-toxicity without side effects, sufficient biocompatibility, thermal stability, and a narrow band gab energy of about 2.7 eV and promote the photocatalytic activity under visible light.^21–25^ Low specific surface area (∼50 m^2^ g^−1^) and ultra-fast attraction of electron–hole pairs inhibit the prolonged time of charge carriers in the photocatalytic process. The low valence band potential (*E*_VB_ = +1.5 eV) of g-C_3_N_4_ fails to generate hydroxyl radicals (·OH), which are essential species for the destruction of organic pollutants. The hybridization of wide and narrow band gap energy semiconductors is a promising issue for successfully generating heterojunctions with appropriate solar radiation absorbability and high separation and transportation efficiency of electron–hole pairs. Silver-based photocatalysts such as AgCl, Ag_3_PO_4_, AgIO_4_, AgVO_3_ and Ag_2_CO_3_ exhibit strong photocatalytic reactivity in the decomposition of organic pollutants.^26–31^ Much attention is drawn towards Ag_2_CO_3_ nanoparticles due to their insolubility in water, low toxicity, adjustable bandgap structures and simple synthesis routes.^32–41^ The successful construction of heterojunctions requires the appropriate adjustment of the band energy structure of the two semiconductors to generate charge carriers with an auspicious redox power.^42–44^ Recent research studies have focused on coupling Ag_2_CO_3_ with g-C_3_N_4_ to generate a successful heterojunction for removing organic containments from wastewater.^45–51^ Konglin *et al.* reported the successful photodegradation of Rhodamine B and methylene blue dyes over g-C_3_N_4_/Ag_2_CO_3_ containing 3.5 wt% g-C_3_N_4_, which was ascribed to the predominant role of g-C_3_N_4_ in limiting the electron–hole recombination.^45^ Lei *et al.* synthesised g-C_3_N_4_/Ag_2_CO_3_ containing 25 wt% g-C_3_N_4_*via* a sonochemical route for destructing RhB dye under a 300 W xenon lamp, and the experimental results manifested that g-C_3_N_4_ increases the life time for charge carriers, which is responsible for the degradation process.^46^ Shugang *et al.* prepared Ag_2_CO_3_/g-C_3_N_4_ containing 40% Ag_2_CO_3_ by a co-precipitation process for removing methyl orange and methylene dyes. The remarkable reactivity of the nanocomposite was ascribed to the influence of Ag_2_CO_3_ in enhancing the electron–hole transportation and separation.^48^ Yun Feng *et al.* prepared Ag_2_CO_3_/g-C_3_N_4_ containing 30% Ag_2_CO_3_ by a precipitation method for the removal of methyl orange and methylene blue. The exceptional reactivity is attributed to the role of Ag_2_CO_3_ in enhancing the efficiency of charge carrier separation. Previous research studies concentrated on doping silver carbonate as a major constituent with g-C_3_N_4_, which is considered a high-cost route for water treatment. In this work, we made an attempt to construct S-scheme Ag_2_CO_3_/g-C_3_N_4_ with a minimum amount of silver carbonate. The previous research explores the construction of Ag_2_CO_3_/g-C_3_N_4_*via* type (II) heterojunctions and direct Z-scheme mechanism. The type (II) heterojunction fails from dynamic, thermodynamic and energetic points of view to explain the actual charge transportation. The electron transfer from high to low conduction band is accompanied by repulsion force between the existing and the transferring electrons. Concurrently, the electron diffusion from high to low energy level dissipates electrons with a strong reducing power. Direct Z-scheme fails to explain the precise analysis of charge migration between the two semiconductors. The construction of S-scheme Ag_2_CO_3_/g-C_3_N_4_ heterojunctions has not been explored in previous research studies. Hybridizing Ag_2_CO_3_ with high positive oxidative potential and g-C_3_N_4_ with high negative reductive potential generate successful S-scheme heterojunctions. The Fermi level and conduction band of g-C_3_N_4_ is higher than those of Ag_2_CO_3_; however, the work function of Ag_2_CO_3_ is greater than that of g-C_3_N_4_. Upon light illumination, the Fermi levels of Ag_2_CO_3_ and g-C_3_N_4_ jump upward and downward until the two Fermi levels are contacted at the interface region. At this contact point, the electrons and holes with low redox potential are attracted toward each other, and vanished leaving a strong internal electric field. Concurrently, the holes and electrons in the higher valence and conduction bands with a strong redox power are consumed in the photocatalytic process. The production of S-scheme systems is more favorably engineered *via* a solution-based approach to control and tune the particle structure and pore matrix. The sonochemical route is a professional solution process for the synthesis of Ag_2_CO_3_/g-C_3_N_4_ heterojunctions with the homogeneous location of Ag_2_CO_3_ on g-C_3_N_4_ active centers. The as-synthesized heterojunction was subjected to decomposition of Rhodamine B under solar irradiation to utilize UV, visible and near infra-red radiations. Exclusively, NIR light elevates the reaction temperature through the photothermal effects, which enhances the photocatalytic activity. In this work, we aimed to synthesise g-C_3_N_4_ from microcrystalline urea *via* a thermal decomposition process. Ag_2_CO_3_ nanoparticles were hybridized with g-C_3_N_4_ sheets in an ultrasonic bath of 300 W intensity. The physicochemical properties of the solid specimens were characterized by XRD, FTIR, HRTEM, EDX, XPS, mapping, N_2_ adsorption–desorption isotherm, DRS and PL analyses. The photocatalytic activity of the heterojunctions was explored by following the degradation of Rhodamine B dye under natural sunlight radiation of 500 W intensity. The key role of the oxygen radicals and charge carriers was elaborated by carrying out various trapping scavenger experiments and following the PL analysis of terephthalic acid as a probe radical material. On the basis of DRS, PL and trapping scavenger analyses, a proposed mechanism for the transportation of charge carriers between Ag_2_CO_3_ and g-C_3_N_4_ semiconductors in the circuit of the heterojunction is illustrated.

## 2. Materials and methods

### 2.1. Material

Isopropanol, urea, sodium carbonate, methanol, silver nitrate, benzoquinone, ammonium oxalate, Rhodamine B dye and terephthalic acid were supplied by Sigma-Aldrich Company.

### 2.2. Preparation of g-C_3_N_4_ nanosheets

g-C_3_N_4_ sheets were synthesized from urea after purification *via* recrystallization by 200 mL of methanol (99%) with continuous stirring at 150 °C. The solution was allowed to stand at room temperature to get rid of volatile methanol carefully. Then, 100 g of recrystallized urea was annealed in a reactor made of aluminum metal at 520 °C for 3 hours and the obtained solid was washed with distilled water and dried at 80 °C. Finally, a yellow solid specimen was obtained and ground in a porcelain mortar to generate g-C_3_N_4_ nanosheets.

### 2.3. Preparation of Ag_2_CO_3_ nanoparticles

First, 6 g AgNO_3_ dissolved in 100 mL distilled water was added to a solution containing 2 g Na_2_CO_3_ dissolved in 100 mL distilled water with continuous stirring for 1 hour to ensure homogenous distribution. The solution was sonicated for 30 min in an ultrasonic bath at 300 W intensity followed by filtration, washing with distilled water and drying at 110 °C. A dark yellow solid was obtained, ground and stored in a falcon tube.

### 2.4. Preparation of Ag_2_CO_3_/g-C_3_N_4_ nanocomposites

**Fig. 1:**
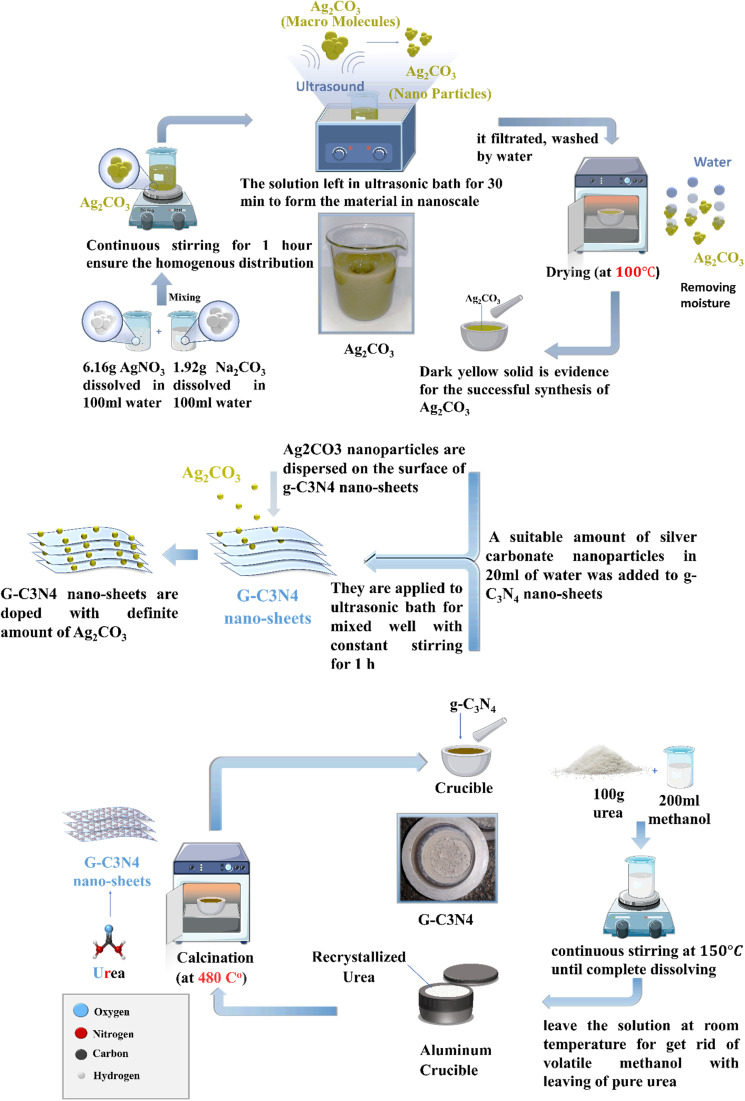
Scheme for the synthesis of Ag_2_CO_3_/g-C_3_N_4_ nanocomposites.

Fig. 1 illustrates the plausible scheme for the synthesis of S-scheme Ag_2_CO_3_/g-C_3_N_4_ heterojunctions *via* a sonochemical route. Typically, a definite amount of silver carbonate nanoparticles dispersed in 20 mL of distilled water were sonicated with g-C_3_N_4_ nanoparticles for 30 min by certain ratio to obtain (5, 10 and 20 w/w%) Ag_2_CO_3_/g-C_3_N_4_. After a while, each of the solution mixture was stirred for 1 h, filtered, washed with distilled water and dried at 80 °C. The photocatalysts are denoted as g-C_3_N_4_, Ag_2_CO_3_, CNAg5, CNAg10 and CNAg20 for pristine g-C_3_N_4_, pristine Ag_2_CO_3_ and the heterojunctions containing 5, 10 and 20 wt% Ag_2_CO_3_, respectively.

### 2.5. Material characterization

A PANalytical X’PERT MPD diffractometer with Cu [Kα_1_/Kα_2_] radiation was employed to investigate the crystalline properties of the as-synthesized heterojunctions. FTIR spectroscopy revealed the functional groups of the nanocomposite samples. Adsorption isotherms of N_2_ at 77 K precisely investigated the surface parameters and pore structure of the solid specimens. HRTEM (JEOL 6340) visualized the particle size distribution of Ag_2_CO_3_ on the sheets of g-C_3_N_4_. A K-ALPHA (Themo Fisher Scientific, USA) instrument with monochromatic X-ray Al Kα radiation in the range of 10–1350 eV was used for XPS analysis. JASCO spectroscopy (V-570) was performed to analyze the diffuse spectrum of the photocatalyst and determine the band energy structure. A lumina fluorescence spectrometer (Thermo Fisher Scientific) was used to analyze the efficiency of photogenerated electron–hole separation.

### 2.6. Photocatalytic degradation of RhB dye

Typically, 0.1 g of the photocatalyst is mixed with 100 mL of 10 mg L^−1^ RhB dye in a solar reactor. The suspension was continuously stirred for 1 hour in the darkness until adsorption/desorption equilibrium was reached. After a while, the mixture solution was exposed to natural sunlight (450 W) at about 3 p.m. (Aug. 2021) for two hours. Then, 4 mL of the mixture was collected at definite time intervals, and then the photocatalyst was separated by centrifugation at 4000 rpm for 10 min. The photocatalytic degradation progress was followed by tracing of the reduction of dye color intensity using a UV-visible spectrophotometer. The photocatalytic mechanism and nature of reactive species were investigated using free radical scrubbers such as AgNO_3_, isopropanol, ammonium oxalate and benzoquinone for detecting conduction band electrons, hydroxyl radicals, holes and superoxide radicals. The photoluminescence of terephthalic acid was investigated to monitor the production of hydroxyl radicals at an excitation wavelength of 325 nm.

## 3. Results and discussions

**Fig. 2:**
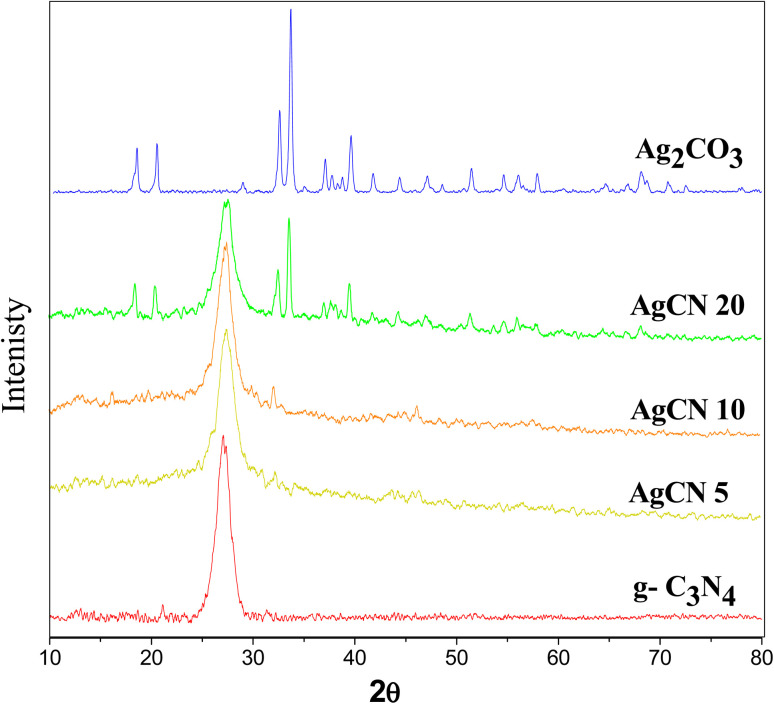
(a) XRD pattern of g-C_3_N_4_, Ag_2_CO_3_, CNAg5, CNAg10 and CNAg20.

**Fig. 3:**
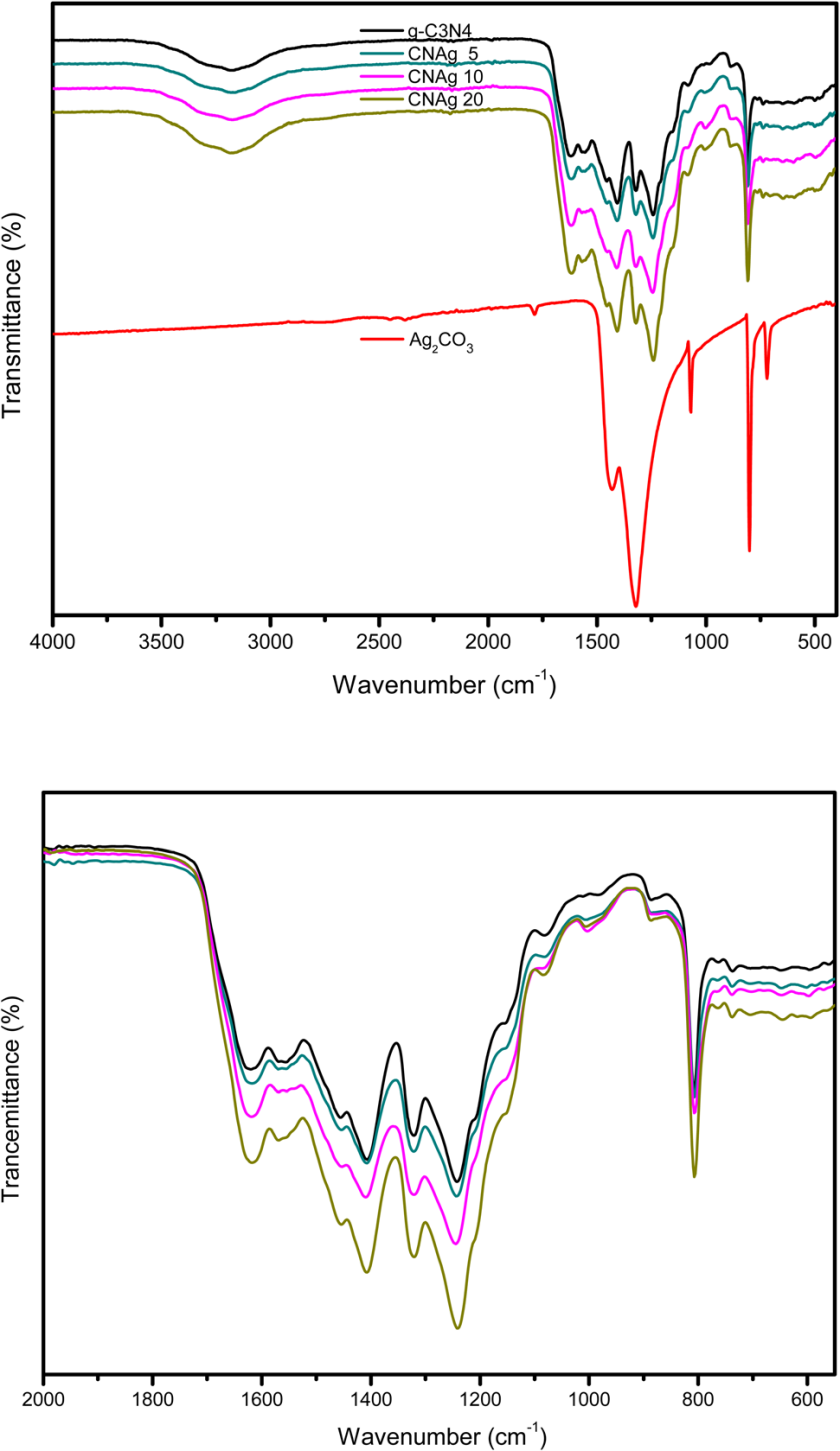
FTIR spectra of g-C_3_N_4_, Ag_2_CO_3_, CNAg5, CNAg10 and CNAg20 nanocomposites.

**Fig. 4:**
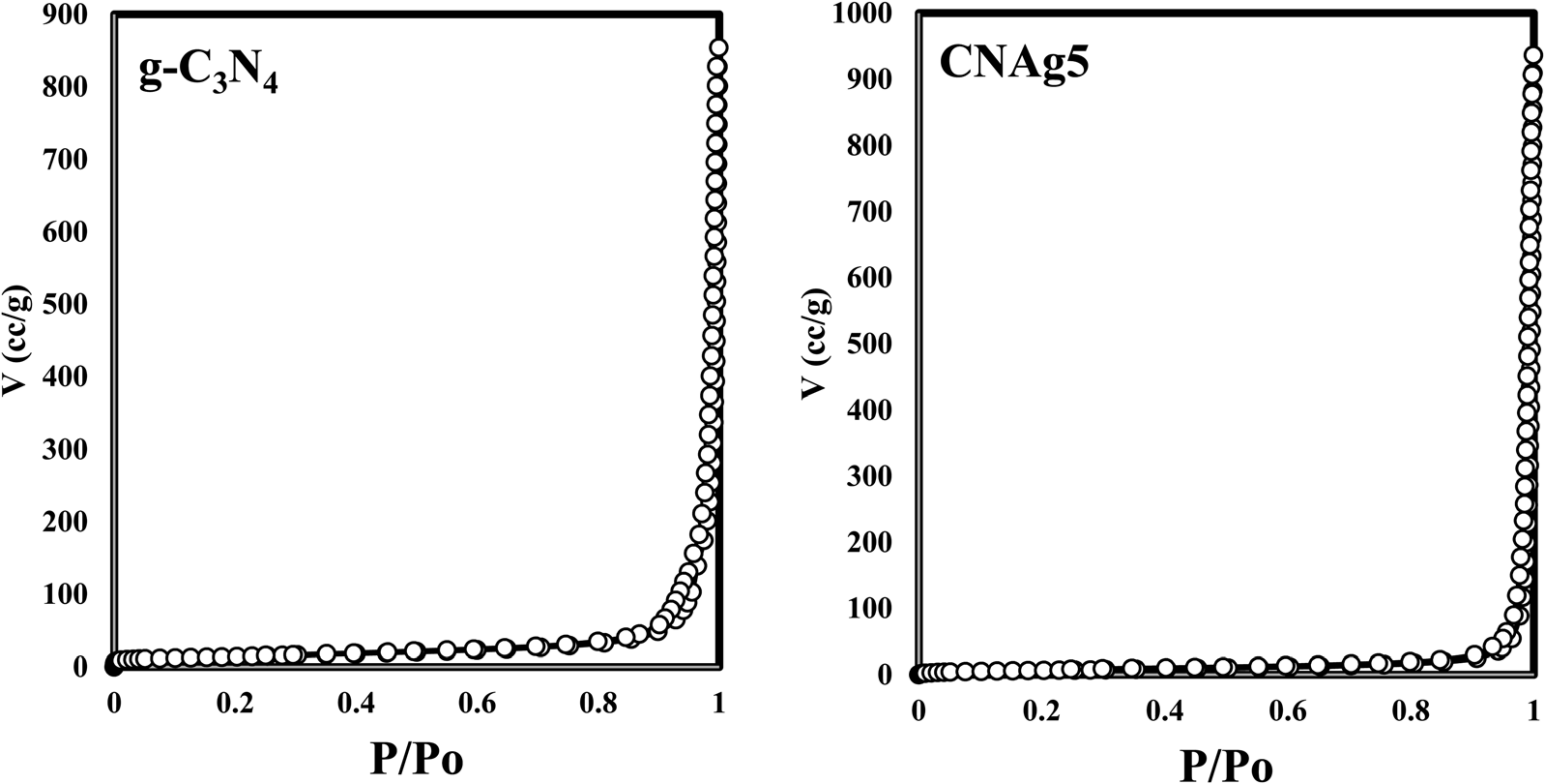
N_2_-adsorption–desorption isotherms of g-C_3_N_4_ and CNAg5.

**Fig. 5:**
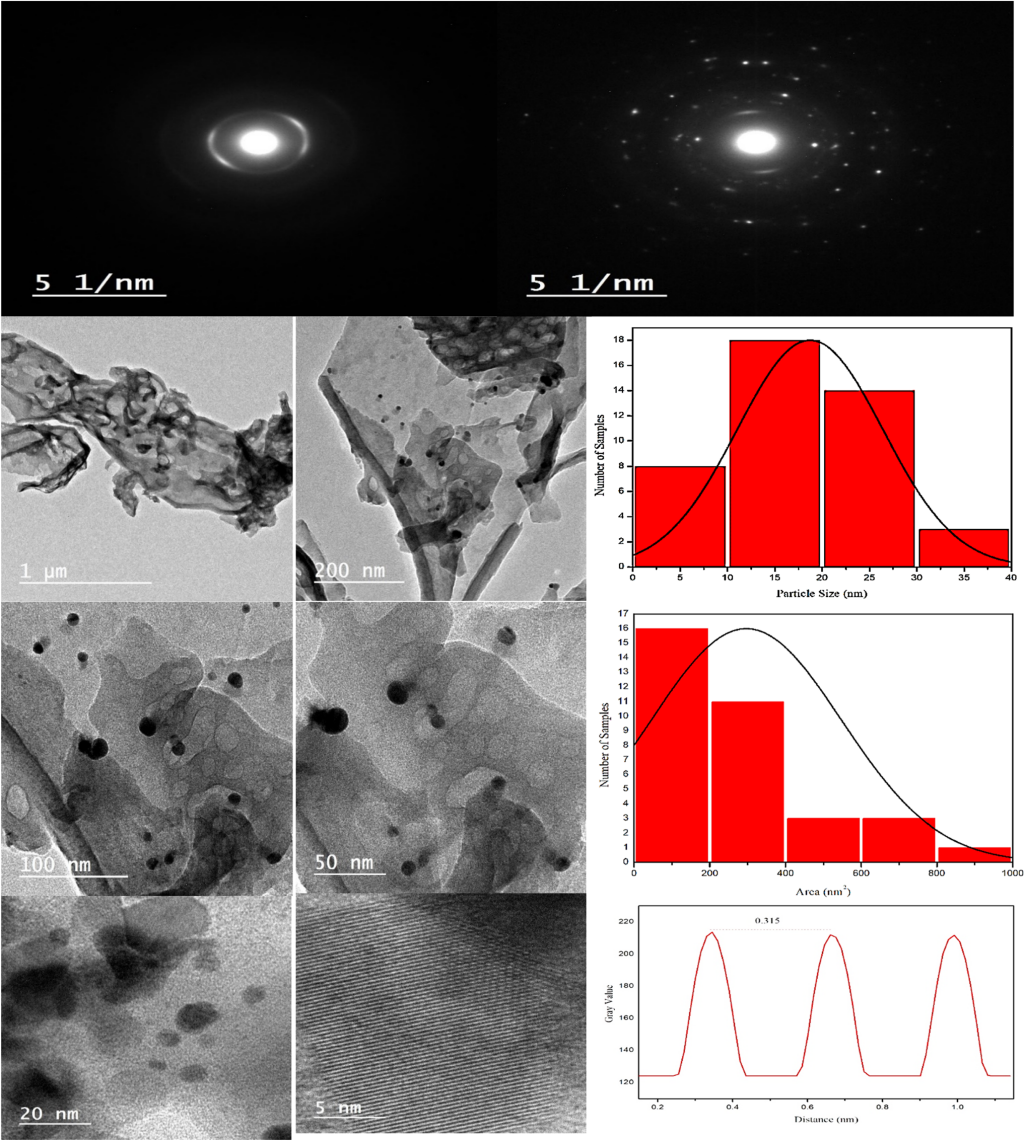
HRTEM image and SAED pattern of CNAg5.

**Fig. 6:**
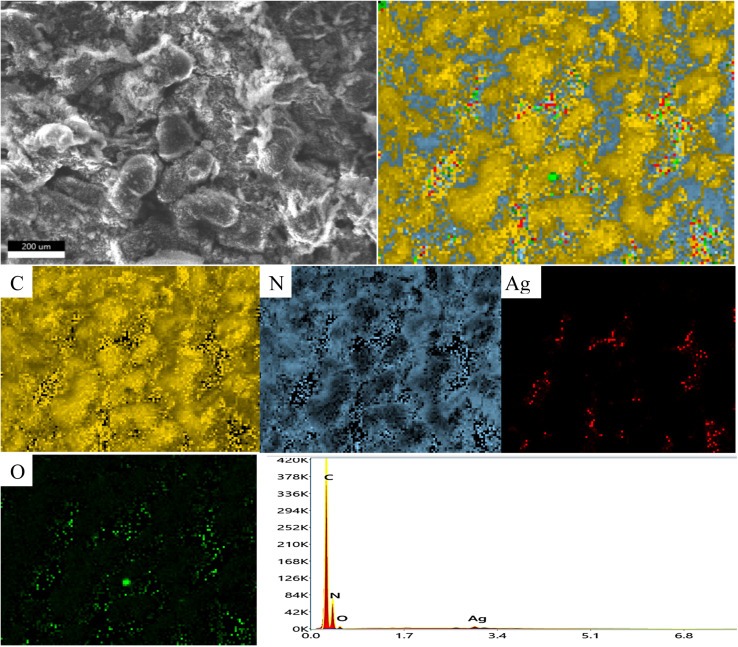
Mapping and EDX spectrum of CNAg5.

**Fig. 7:**
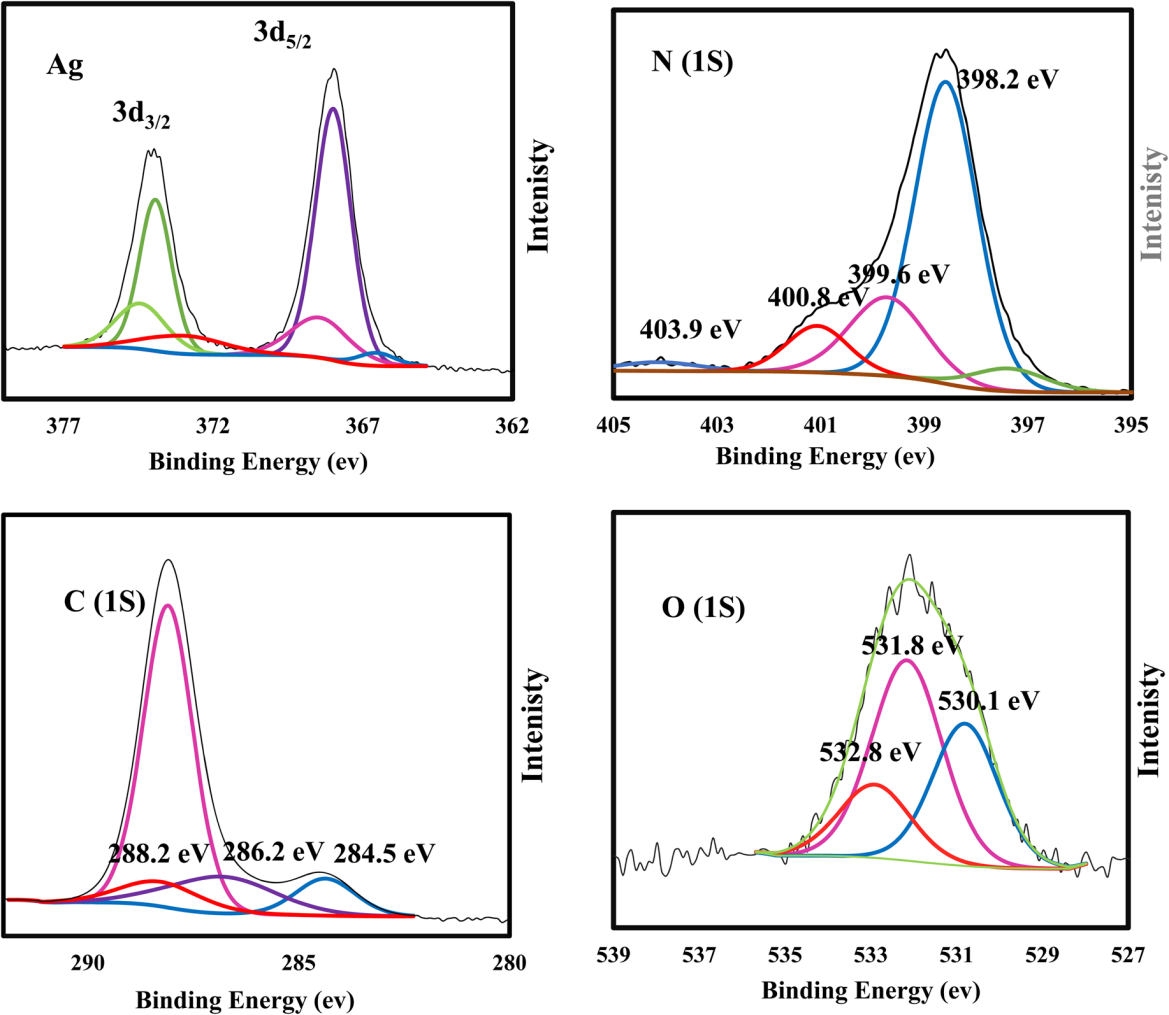
XPS spectra of C, O, N and Ag in CNAg5.

**Fig. 8:**
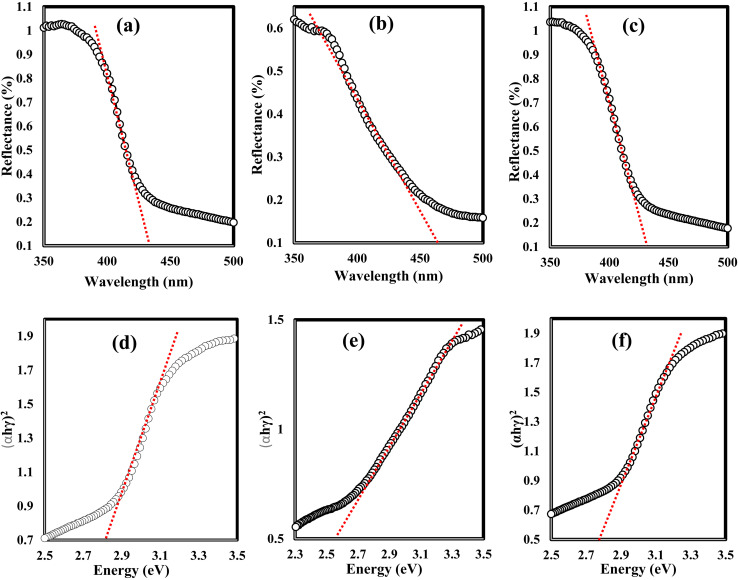
DRS spectra of (a) g-C_3_N_4_, (b) Ag_2_CO_3_, and (c) CNAg5 nanoparticles. Tauc plots of (d) g-C_3_N_4_, (e) Ag_2_CO_3_, and (f) CNAg5 nanoparticles.

**Fig. 9:**
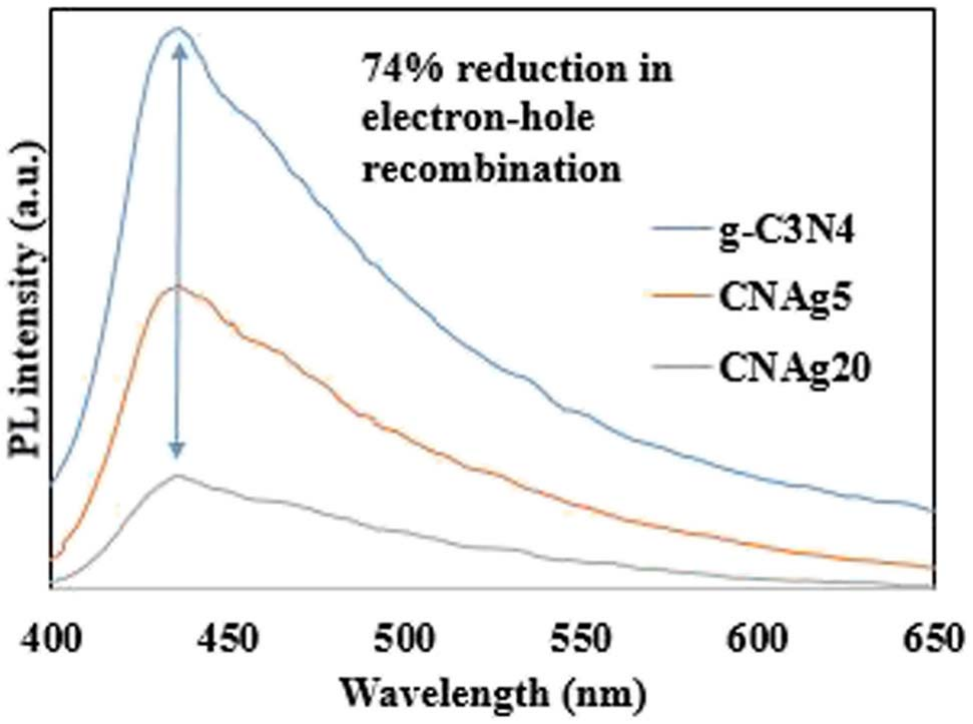
PL spectra of g-C_3_N_4_, Ag_2_CO_3_, CNAg5, CNAg10, and CNAg20.

XRD spectrum explores the crystalline structure of pristine g-C_3_N_4_, Ag_2_CO_3_, CNAg5, CNAg10 and CNAg20 nanocomposites [Fig. 2]. Prevailing diffraction peaks of g-C_3_N_4_ were recorded at 13.2 and 27.59°, which were ascribed to the (100) and (002) diffraction planes (JCPDS no. 87–1526). However, the diffraction peaks recorded at 17.8°, 20.1°, 31.9°, 32.7°, 36.5°, 38.8°, 41.1°, 43.7°, 46.5° and 50.9° were ascribed to Ag_2_CO_3_ (JCPDS No. 23–0339) in the monoclinic crystalline structure. The Debye–Scherrer equation records that the crystalline size is 23.5, 30.7, 8.5, 7.3 and 6.5 nm for g-C_3_N_4_, Ag_2_CO_3_, CNAg5, CNAg10 and CNAg20 nanoparticles, respectively. The XRD pattern of the CNAg5 nanocomposite containing 5 wt% Ag_2_CO_3_ resembled the diffraction pattern of g-C_3_N_4_ with complete missing of the diffraction peaks assigned to Ag_2_CO_3_, revealing the homogeneous dispersion of Ag_2_CO_3_ between g-C_3_N_4_ sheets. The prevailing diffraction peaks assigned to monoclinic Ag_2_CO_3_ were vividly recorded in the nanocomposites containing 10 and 20 wt% Ag_2_CO_3_. The chemical interaction between g-C_3_N_4_ and Ag_2_CO_3_ was investigated by the FTIR spectrum. In Fig. 3a, the spectrum of g-C_3_N_4_ shows various peaks in the range of 1100–1700 cm^−1^ assigned to the stretching vibration of heterocyclic C–N and C=N bonds and the peak at 808 cm^−1^ is attributed to the triazine units, and the broad peak in the range of 3000–3500 corresponds to N–H and O–H bonds of physically adsorbed water. For the spectrum of Ag_2_CO_3,_ four peaks were found at 720, 800, 1320, and 1430 cm^−1^, indicating the presence of the CO_3_^2−^ group. N_2_-adsorption–desorption isotherms of pristine g-C_3_N_4_ and CNAg5 were classified as type (II) with a closed hysteresis loop, which was ascribed to the non-porous structure [Fig. 4]. The surface area of g-C_3_N_4_ and CNAg5 is 55.5 and 44.3 m^2^ g^−1^ according to the BET equation in its normal range of applicability. The existence of such open surface prohibits the photocatalytic reaction with unrestricted accommodation of pollutant molecules. However, porous systems restrict the diffusion and transportation of RhB molecules due to the pore constrictions. The HRTEM image of the CNAg5 heterojunction is represented in Fig. 5, which records the generation of g-C_3_N_4_ sheets with laminar structure containing various wrinkle points. On careful exploration of HRTEM images at different magnifications, one can notice the successful deposition of spherical Ag_2_CO_3_ nanoparticles on the active sites of g-C_3_N_4_ sheets at equidistant positions in the homogeneous arrangement, which reveals the strong chemical interaction between Ag_2_CO_3_ nanoparticles and g-C_3_N_4_ sheets. The particle size distribution was elucidated by constructing histograms that reveal that major nanoparticles exhibit a size varying between 15 and 20 nm. HRTEM records the existence of lattice fringes of spacing 0.315 nm, which were ascribed to the (100) plane of Ag_2_CO_3_. SAED analysis manifests the existence of different rings ascribed to the crystalline planes of g-C_3_N_4_ and Ag_2_CO_3_ nanoparticles. Mapping and EDX elemental analysis results are illustrated in Fig. 6, revealing the uniform distribution of C, N, Ag and O in the CNAg5 heterojunction. The production of porous graphitic carbon nitride sheets with spongy structure was clearly observed with the homogeneous distribution of Ag_2_CO_3_ on the localized active sites on g-C_3_N_4_ sheets. The oxidation state and elemental distribution of the heterojunction constituent was investigated by XPS analysis [Fig. 7]. The spectrum indicates the existence of Ag, O, C and N with a perspective binding energy, revealing the high purity of the nanocomposite without the existence of any contaminant from the preparation medium. At binding energies of 367 eV and 373 eV, Ag (3d) was detected, which deconvoluted into Ag 3d_5/2_ and Ag 3d_3/2_, respectively, having a spin–orbit separation of *ca.* 6.0 eV as detected in the previous research studies. The C 1s signal was resolved into three different peaks at 284.28 eV, 287.68 eV and 293.28 eV, corresponding to the C–C bonding states of surface carbon, sp^2^-bonded C (N–C=N) groups and carbonate (CO_3_^2−^) groups from Ag_2_CO_3_. The broad peak of the N 1s spectra was resolved into four peaks at 398.6, 399.8, 400.4 and 403.9 eV, assigned to C–N–C, N–(C)_3_, C–NH_*x*_, and π excitation, respectively. Moreover, the characteristic peak of O 1s was deconvoluted into three peaks at 530.1 eV, 531.8 and 532.8 eV which were ascribed to the C–O and C=O bonds in Ag_2_CO_3_ and the surface of composite adsorbed –OH groups. The color of the nanocomposite became darker upon the incorporation of various contents of silver carbonate, revealing the shifting in the solid specimen's response to the visible light absorbability. Fig. 8 illustrates the DRS analysis of pristine g-C_3_N_4_, Ag_2_CO_3_ and Ag_2_CO_3_/g-C_3_N_4_ heterojunctions. The band gap energy was calculated according to the Tauc equation to determine the type of the electronic transition. The shifting in light absorbability of g-C_3_N_4_ from 420 to 470 nm is sufficient to enhance the photocatalytic reactivity of the solid specimen in the visible light region. The valence and conduction band potentials were calculated on the basis of band gap energy and the electronegativity of g-C_3_N_4_ and Ag_2_CO_3_. The valence and conduction band potentials of g-C_3_N_4_ are +1.57 and −1.13 eV. However, the valence and conduction band potentials of Ag_2_CO_3_ are +2.55 and +0.45 eV. The electron–hole separation efficiency for single-phase g-C_3_N_4_ and the as-synthesized heterojunctions was explored by constructing the PL spectrum of solid specimens [Fig. 9]. A fantastic emission signal at an intensity of 445 nm belongs to the electron–hole coulombic attraction recorded in the PL spectrum. About 40, 45, 68 and 73% reduction in the PL peak intensity was detected with the introduction of 5, 10, 15 and 20 wt% Ag_2_CO_3_ on the g-C_3_N_4_ surface. These results clearly indicated the significant role of Ag_2_CO_3_ in decreasing the electron–hole coulombic attraction force as well as promoting strong interactions between Ag_2_CO_3_ and g-C_3_N_4_.

## 4. Photocatalytic degradation of Rhodamine B dye

**Fig. 10:**
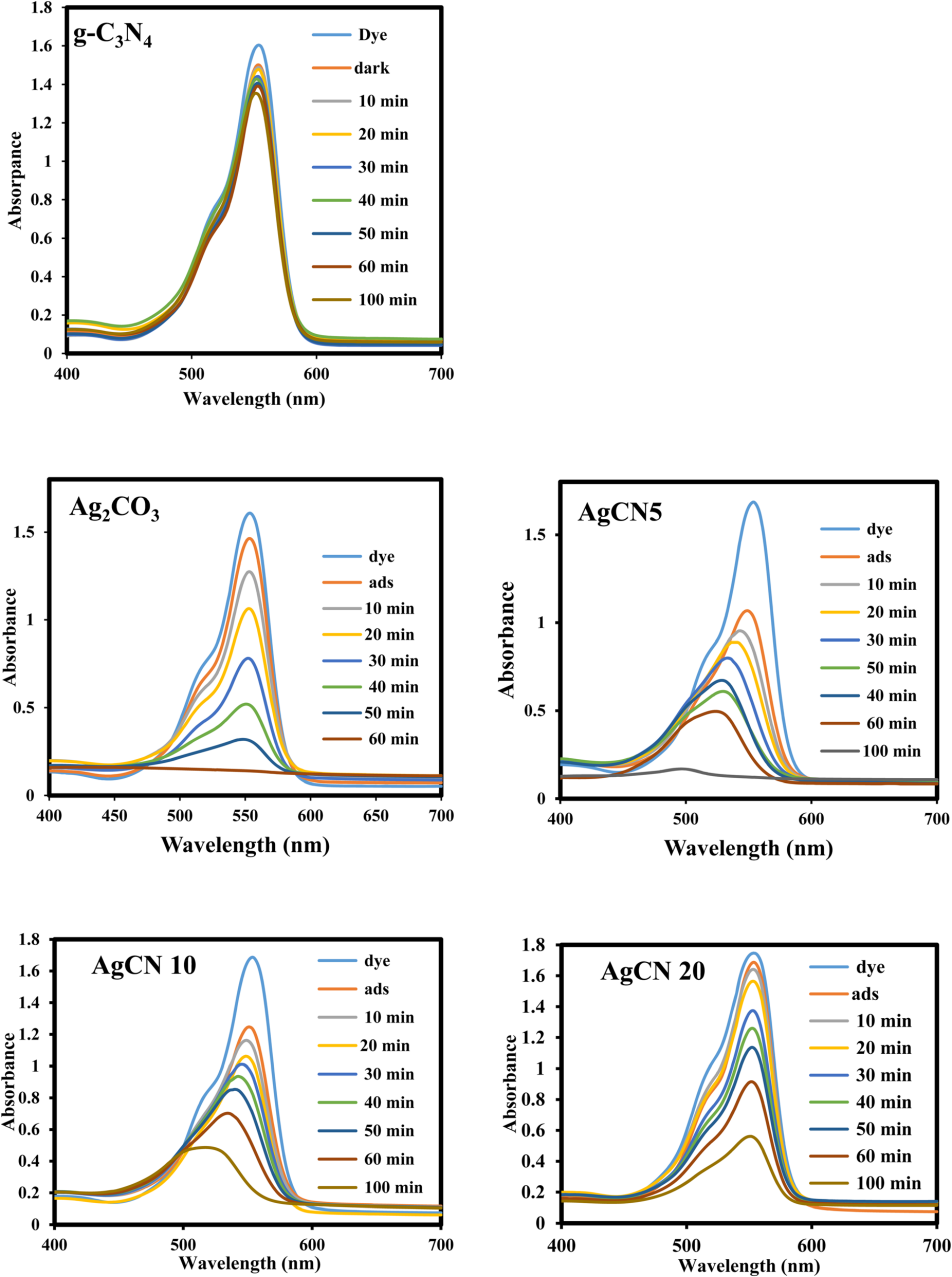
Absorption spectrum of photodegradation of Rhodamine B dye over g-C_3_N_4_, Ag_2_CO_3_, CNAg5, CNAg10 and CNAg20.

**Fig. 11:**
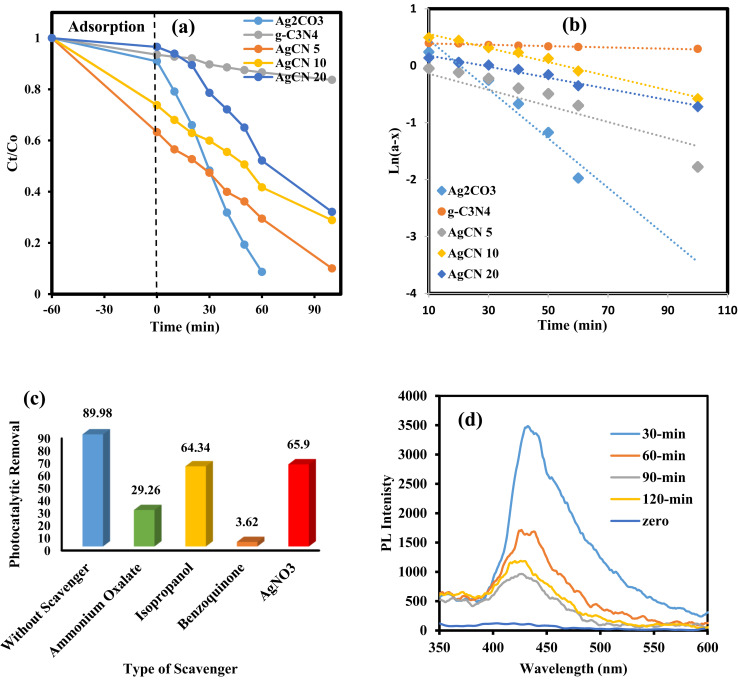
(a) Variation in the removal of RhB dye over the surface of g-C_3_N_4_, Ag_2_CO_3_, CNAg5, CNAg10 and CNAg20 and the time of irradiation. (b) Pseudo-first order plot for the photodegradation of RhB over the surface of g-C_3_N_4_, Ag_2_CO_3_, CNAg5, CNAg10 and CNAg20. (c) Effect of various scavengers on the photocatalytic degradation of RhB dye over CNAg5 nanocomposites. (d) PL spectrum of terephthalic acid at 315 nm excitation wavelength over CNAg5 nanocomposites against the time of irradiation.

The ubiquitous existence of dyes and antibiotics in wastewater is an alarming environmental issue that affects human health and aquatic life. Among the industrial dyes, Rhodamine B is one of the most employed dyes in industries. Rhodamine B dye (RhB) is toxic, carcinogenic, and non-biodegradable that poses a serious threat to human health. Various routes were developed for the safe destruction of RhB dye into eco-friendly species without producing a secondary generation of pollutants. Photocatalytic destruction of RhB under natural solar irradiation into CO_2_, water and small molecules is an auspicious route for expelling RhB dye from wastewater. The efficiency of the as-synthesized heterojunctions in degrading RhB was tested and compared under natural sunlight irradiation [Fig. 10] to investigate the influence of change in silver carbonate concentration on the efficiency of g-C_3_N_4_ sheets. The decomposition of Rhodamine B dye under natural sunlight irradiation in the absence of photocatalysts is negligible due to the strong chemical stability of the dye solution. Pristine g-C_3_N_4_ sheets exhibit poor efficiency in degrading RhB dye due to the ultra-fast recombination rate of electron–hole pairs and mild absorption responsibility under solar irradiation. On the contrary, pristine Ag_2_CO_3_ shows high efficiency in degrading RhB dye, which results from the matching in band gap energy with sunlight absorbability. However, the high cost of the silver precursor hampers the industrialization of Ag_2_CO_3_ for wastewater treatment. The incorporation of small amounts of Ag_2_CO_3_ on the surface of low-cost semiconductors is a promising route for the manipulation of efficient heterojunctions with a strong redox power. Compared with pristine g-C_3_N_4_, the heterojunctions containing 5, 10, and 20 wt% recorded a fast rate of dye decomposition that reached 95% of the dye initial concentration on the surface of heterojunctions containing 5 wt% Ag_2_CO_3_ [Fig. 11a]. The decomposition of RhB dye passes through several intermediates generated by the expelling of the ethyl groups one by one. This process is trusted by observing various absorption peaks at different wavelengths arranged in descending order. The process of de-ethylation of the fully *N*,*N*,*N*^−^,*N*^−^-tetra-ethylated Rhodamine molecule generates different intermediates as *N*,*N*,*N*^−^-tri-ethylated Rhodamine, *N*,*N*^−^-di-ethylated Rhodamine, and *N*-ethylated Rhodamine at 540, 522 and 502 nm, respectively. The rate of pseudo first order of the dye degradation is 0.0012, 0.0434, 0.0141, 0.0123 and 0.0098 over g-C_3_N_4_, Ag_2_CO_3_, CNAg5, CNAg10 and CNAg20, respectively [Fig. 11b]. The kinetic results indicated that the rate of dye degradation over CNAg5 is fourfold higher than that on pristine g-C_3_N_4_. Scrubber trapping experiments using benzoquinone, isopropanol, ammonium oxalate and silver nitrate were carried out to explore the role of superoxide and hydroxyl radicals besides the positive hole and electron conduction band on the destruction of RhB dye. The experimental result recorded a preferential retardation in RhB dye mineralization in the presence of benzoquinone, isopropanol and ammonium oxalate, which directed the attention toward the positive role of reactive oxygen species and positive hole in the photocatalytic reaction [Fig. 11c]. The production of OH˙ groups under solar irradiation on the CNAg5 surface was recorded by following the intensity of PL emission signals of hydroxyl terephthalic acid at 424 nm [Fig. 11d]. The peak intensity elevates with the increase in the irradiation time, which is taken as evidence for increase in the production of OH˙ species.

**Fig. 12:**
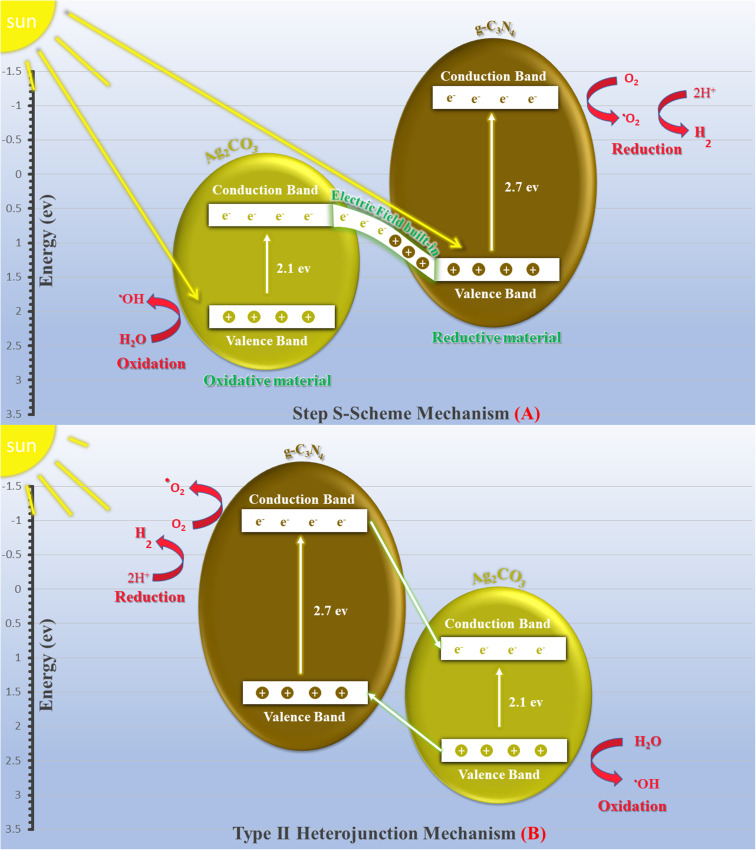
Scheme for the charge transfer between g-C_3_N_4_ and Ag_2_CO_3_ under natural solar irradiation adopting (a) Step S-scheme mechanism and (b) Type (II) heterojunction.

The exceptional photocatalytic reactivity of the as-synthesized samples was attributed to the construction of S-scheme Ag_2_CO_3_/g-C_3_N_4_ heterojunctions. This novel heterojunction harvests the full broad spectrum and enhances the efficiency of electron–hole pair separation and transportation under solar irradiation. The S-scheme heterojunction is composed of a Ag_2_CO_3_ oxidative photocatalyst and a g-C_3_N_4_ reductive photocatalyst. Upon light illumination, electrons are transferred from g-C_3_N_4_ with a greater Fermi level and more negative conduction band potential to Ag_2_CO_3_ with a low Fermi level [Fig. 12]. Concurrently, the Fermi levels of Ag_2_CO_3_ and g-C_3_N_4_ photocatalysts jump upward and downward until the two Fermi levels are equalized. The fruitless holes and electrons of g-C_3_N_4_ and Ag_2_CO_3_ respectively are attracted to each other by coulombic attraction force and vanished. On the contrary, the hot holes of Ag_2_CO_3_ and electrons of g-C_3_N_4_ with a higher redox potential were consumed in the photocatalytic process. This mechanism is manifested from the scrubber experiments and the emission PL analysis of hydroxyterephthalic acid. The holes of the valence band of g-C_3_N_4_ (*E*_VB_ = 1.5 eV) and electrons of the conduction band of Ag_2_CO_3_ (*E*_CB_ = +0.5 eV) are useless charge carriers with a weak redox potential and are attracted toward each other by coulombic attraction force and removed leaving a strong built-in internal electric field. On the contrary, the valence band holes of Ag_2_CO_3_ (*E*_VB_ = +2.5 eV) and electrons in the conduction band of g-C_3_N_4_ (*E*_CB_ = −1.3 eV) were consumed readily in the photocatalytic process. Ag_2_CO_3_ positive holes with a potential of 2.55 eV generate hydroxyl radicals (*E*_OH^−^/OH˙_ = + 2.4 eV) and electrons in the CB of g-C_3_N_4_ with a potential of −1.13 eV generate superoxide radicals 
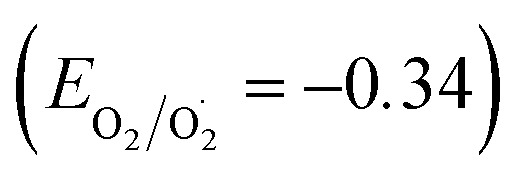
. The classical staggered type (II) heterojunction cannot give a true pathway for charge diffusion between oxidative Ag_2_CO_3_ and reductive g-C_3_N_4_ sheets. The electrons jumping from the conduction band of g-C_3_N_4_ to the conduction band of Ag_2_CO_3_ fail to produce superoxide radicals 
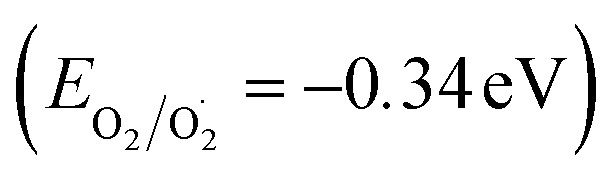
 and the transfer of holes from the Ag_2_CO_3_ valence band to the valence band of g-C_3_N_4_ cannot oxidize water to produce hydroxyl radicals 
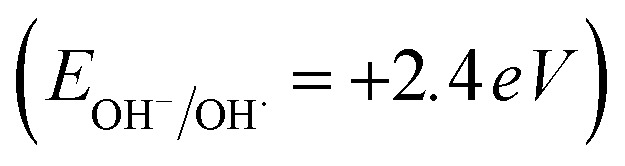
. On the basis of the aforementioned results, the S-scheme heterojunction is the actual mechanism for explaining the precise charge transportation in the Ag_2_CO_3_/g-C_3_N_4_ heterojunction. Table 1 provides the comparative study of the decomposition of various organic dyes on the surface of Ag_2_CO_3_/g-C_3_N_4_ prepared *via* various approaches such as hydrothermal, co-precipitation and sonochemical routes. The results recorded in the previous research studies manifest the requirement of a large proportion of Ag_2_CO_3_ in the nanocomposite, which is a negative economic factor due to the large cost of silver precursors. The mechanism of charge transportation is discussed through the type (II) heterojunction or direct Z-scheme, which fail together in explaining the actual charge migration on the heterojunction surface. On the contrary, our research developed a low-cost and simple sonochemical route for developing Ag_2_CO_3_/g-C_3_N_4_ with 5 wt% Ag_2_CO_3,_ which is efficient to destruct 95% of RhB dye *via* S-scheme heterojunctions.

**Table 1:** Comparative study

Composite	wt%	Preparation	Pollutant	Source of light	Degradation %	Time (min)	Reference
Ag_2_CO_3_/g-C_3_N_4_	5%	Sonochemical	Rhodamine B 100 mL, 10 mg L^−1^	Natural sunlight	Rhodamine B 100%, 10 mg of catalyst	100	Our research
g-C_3_N_4_/Ag_2_CO_3_	3.5%	Sonochemical	Rhodamine B, methylene blue, 30 mL, 10 mg L^−1^	Xe lamb 500 W	Rhodamine B 100%, methylene blue 100%, 20 mg of catalyst	60	45
g-C_3_N_4_/Ag_2_CO_3_	25%	Sonochemical	Rhodamine B, 100 mL 5 mg L^−1^	Xe lamb 300 W	Nearly 100%, 100 mg of catalyst	40	46
Ag_2_CO_3_@/g-C_3_N_4_ core-shell	5%	Co-precipitation	Rhodamine B, methylene blue, methyl orange, phenol, 100 mL, 10 mg L^−1^	250 W halide lamp	Rhodamine B above 95%	54	47
					Methylene blue 96%	54	
					Methyl orange 96.7%	54	
					Phenol 85.5%, 0.1 g of catalyst	40	
Ag_2_CO_3_/g-C_3_N_4_	40%	Co-precipitation	Methyl orange, methylene blue, 50 mL 10 mg L^−1^	Xe lamb 500 W	Methyl orange 93.9%	180	48
					Methylene blue 62.8%, 20 mg of catalyst	240	
Ag_2_CO_3_/g-C_3_N_4_	10%	Co-precipitation	Rhodamine B, 250 mL, 5 mg/L	Natural sunlight	100%, 0.1 g of catalyst	30	49
Ag_2_CO_3_/g-C_3_N_4_	7%	Facile precipitation	Methyl orange, 50 mL, 10 mg L^−1^	500 W Xe lamp	90%, dosage of catalyst 1g L^−1^	10	50
Ag_2_CO_3_/g-C_3_N_4_-MN	30%	Facile precipitation	Methyl orange, rhodamine B, 90 mL, 10 mg L^−1^	300 W Xe lamp	Methyl orange, above 95%, 30 mg of catalyst	30	51

## 5. Conclusions

In this research work, S-scheme Ag_2_CO_3_/g-C_3_N_4_ heterojunctions have been generated by hybridizing different concentrations of spherical Ag_2_CO_3_ nanoparticles and g-C_3_N_4_ sheets sonochemically for expelling RhB dye. The experimental analysis indicates that the localized deposition of Ag_2_CO_3_ on g-C_3_N_4_ sheets, deep absorption of solar radiation, better electron hole–electron separation and transportation are the main parameters for optimizing the photocatalytic efficiency of the heterojunction composed of 5 wt% Ag_2_CO_3_ and 95% g-C_3_N_4_ sheets. The destruction of Rhodamine B dye passes through a series of intermediate organic compounds that finally decompose completely into CO_2_ and H_2_O. The charge transportation *via* the S-scheme photocatalytic mechanism was manifested from scrubber trapping experiments and PL analysis of terephthalic acid. Superoxide radicals in addition to positive holes demonstrate a predominant contribution role in degrading Rhodamine B dye under natural sunlight of 500 W intensity.

## Conflicts of interest

There are no conflicts to declare.

## Supplementary Material
